# Securizing data linkage in french public statistics

**DOI:** 10.1186/s12911-016-0366-4

**Published:** 2016-10-06

**Authors:** Maxence Guesdon, Eric Benzenine, Kamel Gadouche, Catherine Quantin

**Affiliations:** 1 CHRU Dijon, Service de Biostatistique et d’Informatique Médicale (DIM), Université de Bourgogne Franche-Comté, Dijon, France; 2 INRIA, Institut National de Recherche en Informatique et Automatique, Palaiseau, France; 3 Centre d’Accès Sécurisé aux Données (CASD), Malakoff, France; 4 INSERM, CIC 1432, Dijon University Hospital, Clinical Investigation Center, clinical epidemiology/clinical trials unit, Dijon, France; 5 INSERM UMR 1181 “Biostatistics, Biomathematics, Pharmacoepidemiology and Infectious Diseases” (B2PHI), Univ. Bourgogne Franche-Comté, Dijon, France

**Keywords:** Data linkage, Patient data privacy, Population statistics

## Abstract

Administrative records in France, especially medical and social records, have huge potential for statistical studies. The NIR (a national identifier) is widely used in medico-social administrations, and this would theoretically provide considerable scope for data matching, on condition that the legislation on such matters was respected.

The law, however, forbids the processing of non-anonymized medical data, thus making it difficult to carry out studies that require several sources of social and medical data.

We would like to benefit from computer techniques introduced since the 70 s to provide safe linkage of anonymized files, to release the current constraints of such procedures.

We propose an organization and a data workflow, based on hashing and cyrptographic techniques, to strongly compartmentalize identifying and not-identifying data.

The proposed method offers a strong control over who is in possession of which information, using different hashing keys for each linkage. This allows to prevent unauthorized linkage of data, to protect anonymity, by preventing cumulation of not-identifying data which can become identifying data when linked.

Our proposal would make it possible to conduct such studies more easily, more regularly and more precisely while preserving a high enough level of anonymity.

The main obstacle to setting up such a system, in our opinion, is not technical, but rather organizational in that it is based on the existence of a Key-Management Authority.

## Background

In the early 1970s, France had very promising statistical potential thanks to the wealth of information in its administrative files, which could be exploited together or in the context of a survey.

In 1974, computerization of the civil status register under the unfortunate name of the SAFARI^1^ project gave rise to considerable public outcry, with the fear that personal information concerning the whole population would be recorded and could be misused in the case of a totalitarian government coming into power. Indeed, this national identity number makes it possible to link information relative to the same person from many administrative files, as is currently done, with great precaution, by the statistics institutes of all North-European countries.

This debate led to considerable reflection on the measures necessary to safeguard the privacy and freedoms of individuals in the face of increasing computerization. It gave rise to the law ‘Informatique et libertés’ voted on the 6th January 1978, which established CNIL^2^.

The impact of this law was globally very positive. However, it blocked the use of administrative statistics, either by limiting statistics to the processing of a single file, or, when crossing two or more files, by imposing procedures that were disproportionately heavy and have in the past proved to be strongly dissuasive (such as the requirement for a decree from the Council of State).

In order to avoid the dangers of the general linkage of administrative files, CNIL opted for a strategy that made it impossible to use the same identifier in every file. As the NIR^3^ was already widely used in social and healthcare administrations, the CNIL restricted its use to the sectors of work, healthcare and social institutions. For the other sectors (finance^4^, education, …), new so-called “sectorial” identifiers were created, with no possibility of linking the NIR to these new identifiers, even though such links would have been useful for public statistics or research purposes. Today, secure linkage techniques make it possible to overcome these restrictions. This is the subject of this article.

The law forbids the manipulation of non-anonymized personal medical data. However, it would be impossible to link files containing personal medical data if these data have been anonymized in the strictest sense, since there is no information that would make it possible to link data from two different files for one individual. Henceforth, we will use the term anonymization to speak about relative anonymization, which is notably based on pseudonymization. Pseudonymization consists in systematically replacing each value of an identifier field with another value, with no possibility of returning to the initial value.

Our article, which is limited to pseudonymization of the identifier, does not cover indirect identification, which is all the more likely the more different files are linked. Of course, this risk of identification depends on the information, certainly unknown, held by the ill-intentioned third party. We are thus talking about a “degree of anonymization”, or “more-or-less partial anonymization”, which is not really provided for in the law.

Since the 1970s, computer techniques have also evolved. Current techniques now make it possible to safely link data from different institutions while preserving anonymity thanks to hashing (cf. section Hashing) of the common identifier or, in the absence of such an identifier, by probabilistic linkage (cf. section Probabilistic linkage).

In section A statistical information system, we expose a strategy to safely overcome the constraints described above, inspired from the one proposed by Quantin for epidemiology [1], and we propose to apply this strategy to public statistics so as to increase flexibility without jeopardizing security.

## Methodology

### Hashing

#### Principles

Hashing techniques [2] are computer procedures that consist in calculating a fixed-size *fingerprint* (or signature) from any data whatever the size.

A hash function allows any data to be mapped to an element of a finished set, whose cardinal is very large^5^. This means that there is no inverse operation that will allow the initial data to be retrieved from the fingerprint, because an infinite amount of data have the same fingerprint.

Moreover, the *distance* between two fingerprints of two data is independent of the distance between these data. A minimal difference between two data leads to two very different fingerprints (so-called “avalanche effect”). In contrast, two very different data may have similar or identical fingerprints.

For example, hashing the chains “Dupont” and “Dupond” by the SHA256 function gives the following fingerprints:

SHA256(~Dupont~) = 3bde3a5999601d8fa7b6bcc6bfdd2ee6a9fb473043d9768fbf8274b5936ef4d2

SHA256(~Dupond~) = 535a7594e59be910df06483d24371c7697854fa84d8ed8c0f400126edc25af3a

A good hash function presents a low *risk of collision*, which means that for different data of a similar size, the probability of having the same fingerprint is extremely low^6^. Collisions that could be introduced by hashing are minor compared with problems of homonymity, which can occur in practice when cleartext data such as surnames, first names and dates of birth are manipulated.

As the hash function has no inverse function, processing by hashing is said to be irreversible. However, if the hash function is known, it is possible to retrieve an original data from its signature, thanks to so-called *dictionary attacks*.

#### Resisting attacks

The principle of these dictionary attacks is the following: if one knows the hash function used, one can apply it to a set of chains of characters. One can thus construct a table of correspondence between each chain of characters and its fingerprint from the hashing process.

The rate of collisions (*i.e.* two different chains giving the same fingerprint) of hashing algorithms is extremely low. Thus, to know a particular fingerprint, one simply has to look at the table of correspondence to identify with a high degree of certainty the initial chain of characters.

This type of attack thus poses a problem of data confidentiality if hashing is used to anonymize (or rather pseudonymize) personal data. The solution therefore consists in modifying the chain before applying the hash function. A classical way of proceeding is to add a *salt*, that is to say a secret key to each data before calculating its fingerprint. If, for example, our key is “XZ!#45”, this key is added at the start or the end of the chain to be hashed:

SHA256(~DupontXZ!#45~) = cd0c6a7852dc50474778d2599a6bf85d5c8c1f31a6c4e348a52e4fcd04b8d660

SHA256(~DupondXZ!#45~) = 7e20b3c86d4c1508f1c4b7650ffa62e3fd379bb10fad9b3c618449cb9088d0d0

If neither the size nor the content of the key is known, dictionary attacks become impossible in practice, because, even if one assumes that the size of the key is between 1 and 20 bytes^7^, it is necessary to construct tables of correspondence, which poses a problem of computation time and storage space.

Another way to proceed is to apply a function to the chain. This can be a secret function (or a function using a secret key) that can be used to either modify the chain to be hashed, or to compute a different salt for each chain^8^.

There are several “standard” hashing functions [3] (MD5, SHA1, SHA256, SHA512, …), which are continually being studied for their resistance to attacks. The ANSSI now recommends [4] using the SHA-256 method.

#### Utilization for data linkage

Hashing techniques applied to identifiers make it possible to pseudonymize files to be linked. However, this pseudonymization does have drawbacks.

Indeed, if the slightest error is made in entering the name, for example, the signature for the misspelled name will be completely different from that obtained for the correct name. Upstream normalization procedures may limit such problems [5] (SOUNDEX, lower case only, suppression of accents, …). For the same reason, it is not possible to calculate an edit distance (for example, Levenshtein distances, or Hamming distances, …[6]) to use in deterministic linkage^9^ (cf. section Deterministic linkage).

Hashing is already used to link data from several files in order to safeguard relative anonymity [7].

It is also worth mentioning the use of double hashing when files to be linked come, for example, from several establishments. It is necessary to hash the identifier fields in the same way (with the same secret key) in all of the files to allow linkage according to these fields. However, the establishment that receives these files carries out a second hashing (with a second secret key) so as to render the aggregated data anonymous vis-a-vis the establishments that produced the files [8].

Finally, given the irreversible nature of hashing, it is important to keep the unhashed data, as they may be exploitable only with data that underwent the same hashing process. This implies that the keys used must be managed carefully, with, for example, one key per study, or it may be necessary to set up a Key-Management Authority (cf. section Discussion).

### Deterministic and probabilistic linkage

There are two types of linkage, depending on the data to be linked. See [9] for a review of linkage methods and their use for healthcare data.

#### Deterministic linkage

So-called deterministic linkage consists in determining the identifier fields in the two sources of data to be linked, and then defining a distance and a threshold based on which two records are deemed to belong to the same individual. The term “deterministic” stems from the fact that the thresholds chosen do not depend on the data to be linked, that is to say that the same thresholds are used even if supplementary data are added to the files to be linked.

The classical application of this method consists in deciding to match records for which the identifiers are strictly identical. Thus, by linking data according to the NIR, or a double-hashed NIR, it is easy to carry out this “strict” matching method^10^ and it is as reliable the identifier field used.

In this family of linkage methods, several refinements are possible. One can thus concatenate all of the identifier fields, apply a distance to this concatenation and compare the results with a threshold. One can also apply a different distance to each field so as to obtain a global distance by weighting.

Finally, the distance can also be defined for each field and with a binary result (0 or 1). One can thus define rules that associate a matching decision with each configuration of similitudes and differences between two records.

#### Probabilistic linkage

Probabilistic linkage is useful when there is no unambiguous identifier field (such as the NIR) for the individuals concerned that is common to the two sources of data to be linked, and for which it is impossible to establish rules (for examples, from distances between fields), as is the case when the information has been anonymized by hashing (cf. section Utilization for data linkage).

The probabilistic nature of these methods stems from the fact that they use weights associated with each field used as identifier, and called unit weights. These unit weights depend on the different values present in the fields used as identifiers, their frequency, etc. These unit weights are then summed to obtain a compound weight.

Two thresholds for these compound weights make it possible to classify the pairs of records as “Matched”, “Unmatched” or “Indecision”. These thresholds are chosen in an ad hoc manner, depending on the study and the associated constraints : necessary accuracy, nature and quality of data, tolerance of error due to missing or excess data, possibility to verify and validate, …

Unlike deterministic linkage methods, if data is added to the files to be linked, it will modify the weights used in the linkage decision, and thus the choice of thresholds. The theoretical framework for these probabilistic linkage methods was established in [10] in 1968. In 1995, for the first time Jaro applied these methods to healthcare data in [11] using a computer program. In 1998, [12] described the first application of the Jaro method to files that had been anonymized by hashing.

#### Principles

We seek to link two files, constituted of records, each record being composed of several fields.

The aim is to bring together data for the same patient while minimizing errors :

- Duplicates (false negatives) : not associating two records which concern the same individual. This happens when the information (the fields of records) used to match the records is not precise enough or contains errors (name changes, input errors,...);
- Collisions (false positives) : incorrectly associating information from 2 different people.

Table 1 illustrates these different cases.

**Table 1:** Duplicates and collisions

	Same individual	Different individuals
Same name	True positive	False positive = collision
Different names	False negative = duplicates	True negative

The idea of the method is to take into account the information brought by each value of each field chosen as an identifier (family name, first name, date of birth, …), and its frequency. Thus, the sex will be far less discriminative than the date of birth, because there are in most cases only two possibles values. In the same way, in a file exclusively containing recently new-borns, the year of birth brings little information.

For this reason, a unit weight is attributed to each identity characteristic. The value will be positive in cases when two records correspond and will be negative in cases when two records do not correspond.

The Fellegi and Sunter model proposed to distribute pairs into two sets *M* (for “matched”, the pairs that correspond to the same individual) and *U* (for “unmatched”).

For each identifier field *i*, two probabilities *m*_*i*_ and *u*_*i*_ are calculated. *m*_*i*_ is the probability that the two records have the same value in the field *i* when the pair belong to *M*. *u*_*i*_ is the probability that the two records have the same value in the field *i* when the pair belong to *U*.

Once these two probabilities are known, the unit weight associated with a field will be $log\frac {m_{i}}{u_{i}}$ (positive value) when the values in field *i* correspond, otherwise the weight will be $log\frac {1 - m_{i}}{1 - u_{i}}$ (negative value).

As the pairs that correspond to the same individual are unknown, since this is the aim of the linkage method, these unit weights are estimated thanks to the EM (Expectation-Maximization) algorithm introduced by Winkler [13] or one of its subsequent variants [9, 14]. These algorithms proceed by iteration, by using data to be linked to converge estimators of *m*_*i*_ and *u*_*i*_.

**Fig. 1:**
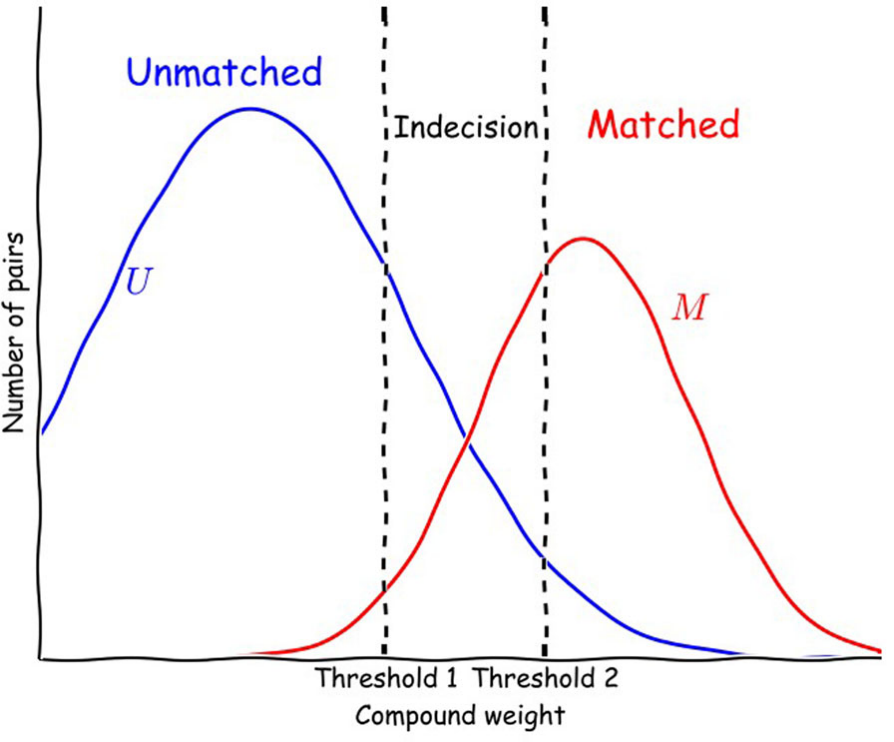
Linkage decision according to compound weight

Once the unit weights have been obtained, they can be summed (they are log likelihood ratios) to obtain a compound weight. The method gives the probabilities that pairs corresponding to each compound weight belong to *M* and *U*. Figure 1 illustrates these probabilities according to compound weight.

We thus obtain an “unmatched” zone, for which the probability of belonging to *M* is low, while the probability of belonging to *U* is high. In another zone “matched”, the probability of belonging to *M* is high while that of belonging to *U* is low. Finally, in the third zone “indecision” it is impossible to decide automatically whether or not the two records concerned can be linked. We therefore have two thresholds.

Depending on the purpose of the linkage, more or less high thresholds can be used to classify each pair of files in these three categories.

#### Example

Let us illustrate this method with a linkage done on hospitalization data of a healthcare establishment (linkage of two successive years), based on three identifier fields: the family name, the first name and the date of birth. Henceforth, the two files to be linked will be called *A* and *B*.

For each field, the unit weight is the same as the log likelihood from Table 1; it is thus additive for all of the individual’s identifier characteristics. Calculation of unit weights, depending on data, gives the results shown in Table 2.

**Table 2:** Results of the unit weight calculation for each of the 3 fields used for linkage

	Family Name	First name	Date of birth
Weight is equal (1)	8.4	5.7	10.3
Weight is different (0)	-2.8	-3.5	-3.1

For each pair of records (one from file *A* and one from file *B*), the compound weight is computed by summing the unit weight associated with each field depending on whether it is the same or different for this field in the two records. Table 3 shows the compound weight of several configurations of equality and differences among the 8 possible configurations (2×2×2) for our 3 fields.

**Table 3:** Computation of the compound weight according to the configuration of equalities and differences

	Family name	First name	DoB	Compound weight
Without disagreement (111)	+8.4	+5.7	+10.3	+24.4
Dis. on the family name (011)	-2.8	+5.7	+10.3	+13.2
Disagreement on the DoB (110)	+8.4	+5.7	-3.1	+11
Disagreement in all fields (000)	-2.8	-3.5	-3.1	-9.4

Table 4 shows the result of a comparison between two records.

**Table 4:** Example of a computation of compound weights for two records

	Family name	First name	Date of birth	
	Dupont	François	29/01/1940	
	Dupont	François	29/03/1940	
Weight	+8.4	+5.7	-3.1	= 11

For each pair, the linkage decision is based on the compound weight thresholds chosen depending on the required accuracy of the study (cf. Table 5).

**Table 5:** Thresholds according to the compound weight

Agreement							
Fam. name	First name	DoB	Frequency	Thresholds	Weight	*P*(*m*)	*G*(*u*)
0	0	0	1 452 966 248		-9.4	6e-08	99.99
0	1	0	4 880 218		-0.2	5e-04	99.99
1	0	0	304 887		1.8	4e-03	99.99
0	0	1	46 081		1.4	0.04	99.96
1	1	0	1 438	“unmatched” threshold	11	28.79	71.21
0	1	1	725		13.2	78.66	21.34
1	0	1	291	“matched” threshold	15.2	96.68	3.32
1	1	1	8 852		24.4	99.99	4e-04

*P*(*m*) : Probability that the 2 records of the pair correspond to the same individual

*G*(*u*) : Probability that the 2 records correspond to 2 different individuals

For the pairs for which there is no automatic decision for linkage, that is to say for the configurations in which the compound weight is between thresholds 1 and 2 (cf. Fig. 1)^11^, manual validation is possible, by returning to the patient’s hospital record^12^. This validation, which allows the linkage or not of “indecision” records, can partly be done automatically by supplementary procedures applied to part of the record pairs, namely those below the automatic linkage threshold, for which a good proportion (for example, as in Table 5, a large part of the 725 pairs agreeing on the family name and the date of birth should be linked; the first name could be different just because of a typing problem). This automated validation can also use other discriminating fields which were not used for automatic classification.

This probabilistic linkage method was notably used to determine vital status by linking hospital data with national mortality data in [15].

In accordance with the legislation, the data had been anonymized beforehand by using the hashing technique. In practice, the comparisons of fields are thus done on hashed data. Thus, Table 4 is more likely to resemble Table 6.

**Table 6:** Example of calculation of compound weights for two anonymized records

	Family name	First name	Date of birth	
	fe1fb20e56bd...	5b7808252fec...	aeed71d1dc67...	
	fe1fb20e56bd...	5b7808252fec...	9b1549d98eab...	
Weight	+8.4	+5.7	-3.1	= 11

#### Linkage by blocks

The application of probabilistic methods to even moderate-sized files requires nonetheless a substantial amount of computation time, due to the cartesian product between the records.

To overcome this problem, the so-called “blocking” method [11, 16] is used. This method makes it possible to match only certain records of file *A* and file *B*. If, for example, the sex field is reliable, we can decide to link only data that match for this field. The same can be done using the year of birth, for example. Blocking can also be done for several fields at the same time (for example, sex and year of birth). Finally, blocks from several fields can be matched successively to find more matches: if the sex field is not reliable, additional matches can be found by using blocking according to the year of birth, or vice-versa.

### Encryption

#### Principles

Encryption techniques [17] (encrypting) consist in making a message unreadable for those who do not have the key to make it readable again. It is a very dynamic field of research, because these techniques lie at the heart of communication security on the Internet, bank transactions, etc.

There are two families of encryption techniques : those that use the same key for encrypting and decrypting (so-called symmetric methods) and those that use two keys, a public key and a private key (so-called asymmetric methods or “public-key” methods).

#### Symmetric methods

In methods that use a single key, the originator and the authorized recipient of the encrypted message must have the same key, which is kept secret. Any person in possession of the key and the encrypted message is able to decrypt the message and thus to get the information it contains.

These methods have several drawbacks. The originator and the authorized recipient must use a secure channel to share the key. In addition, each pair or group of individuals who share secret messages must have the same key reserved for communication with this group of individuals exclusively.

For example, if Alice, Bob and Charlie want to share messages two by two, each of them will need two keys. For each new person they wish to communicate with, each of them will need an additional key, and this without taking into account possible combinations of different compositions of groups of people who may wish to communicate with each other. It quickly becomes difficult to manage all of the keys.

#### Asymmetric methods

Asymmetric encryption methods overcome this problem. These techniques are based on pairs of keys, a public one and a private one. Each person has such a pair of keys. The private key, as its name suggests, is not shared and remains in possession of its owner. The public key, in contrast, can be associated with the owner in the context of an authentification directory, in such a way to ensure that this key is indeed the public key corresponding to the private key of the owner. However, each person can have as many pairs of keys as they wish and share the public part as they see fit.

When a message is encrypted with the private key, only the public key allows it to be decrypted. This means that a message can be signed electronically to authenticate its author^13^. However, it is also possible to encrypt a message with the public key. In this case, only the holder of the corresponding private key can decrypt the message, which ensures the *confidentiality* of exchanges.

Henceforth, we will use the following notations:

- *p* *u* *b*_*X*_ designates the public key of *X* or the public part of key *X*,
- *p* *r* *i* *v*_*X*_ designates the private key of *X* or the private part of key *X*,
- *C*_*k*_(*I*) designates the encryption of information *I* using key *k*; if *k* is a private key, the information will be encrypted for authentication; if *k* is a public key, the information will be encrypted for confidentiality;
- $C^{-1}_{k}(I)$ designates the decryption of the encrypted information *I* using the key *k*.

The following relationships are thus established :

- $C^{-1}_{priv_{X}}\left (C_{pub_{X}}(I)\right) \rightarrow I$ (confidentiality),
- $C^{-1}_{pub_{X}}\left (C_{priv_{X}}(I)\right) \rightarrow I$ (authentication).

It is possible to combine authentication and confidentiality. If Alice wants to send secret information to Bob, while allowing Bob to make sure that this information comes from Alice, Alice will use her private key to sign the message and Bob’s public key to encrypt everything. At reception, Bob will use his private key to decrypt the message and Alice’s public key to make sure that the message is well and truly from her.

Another combination is also possible. For example, if one wants information *I* to become accessible only when two people *A* and *B* agree, it suffices to encrypt this information successively with two public keys. Access to the initial information thus requires the use, in inverse order, of two private keys (one held by *A*, and the other held by *B*) corresponding to the two public keys :

$$\begin{array}{@{}rcl@{}} C^{-1}_{priv_{A}} \left(C^{-1}_{priv_{B}} \left(C_{pub_{B}}(C_{pub_{A}}(I))\right)\right) &\rightarrow& C^{-1}_{priv_{A}} \left(C_{pub_{A}}(I)\right) \\ & \rightarrow & I \end{array} $$

Other combinations are possible, but in the following, we are above all interested in the confidentiality ensured by this encryption system.

#### Utilization for data linkage

These cryptographic methods make it possible to secure exchanges of data, by ensuring both their confidentiality and their origin.

They cannot be used for data anonymization because, unlike hashing, encryption is reversible. However, combined with hashing, they can be used to entrust data linkage to a trusted third party, while separating access to personal data. This use is presented in the following section.

### A statistical information system

The situation is thus as follows : administrations have a wealth of medical, educational and social information, and some of this information uses the NIR as the identifier.

Use of the NIR to link different files requires a decree issued by the Council of State. However, the use of hashing techniques on identifier fields allows the relative anonymization of data. Files with a NIR that have been anonymized using these hashing techniques can be linked without the need for a decree from the Council of State [18, 19].

However, it is not enough to pseudonymize identifier fields (like the NIR) to guarantee a certain level of anonymity. Indeed, while even pseudonymous data can still sometimes be re-identified notably via trajectory information, this risk is even greater when other information is added by linkage. More can be read on this subject in [20].

A trusted third party is thus needed to carry out the linkage and the required statistical studies. This party must have acces to the minimum amount of data necessary for the linkage and the study in question, a study that will moreover require the authorization of CNIL.

In [1], an organization meeting this requirement is proposed for epidemiology. We propose a new organization for public statistics.

#### Principle

Two constraints have to be satisfied. One is the sharing of common identifiers to allow linkage. The other is the constraint of guaranteeing the anonymity of data.

In this section, we will take the example of the NIR as the identifier used for the linkage. The following section will discuss the generalization of this technique to other identifiers.

By applying double hashing to the NIR, it can be used to link files after authorization from CNIL [18]. The hashing keys used to hash the NIR in the two files to be linked must of course be the same. This implies that if an entity is in possession of two files with NIR hashed in the same way, the risk of re-identification is increased.

The idea is to use encryption and hashing of the NIR on the one hand, and trusted third parties on the other, so as to precisely control who has access to what information and who can link this information. To do so, identifying information has to be separated from data, as recommended in [21].

**Fig. 2:**
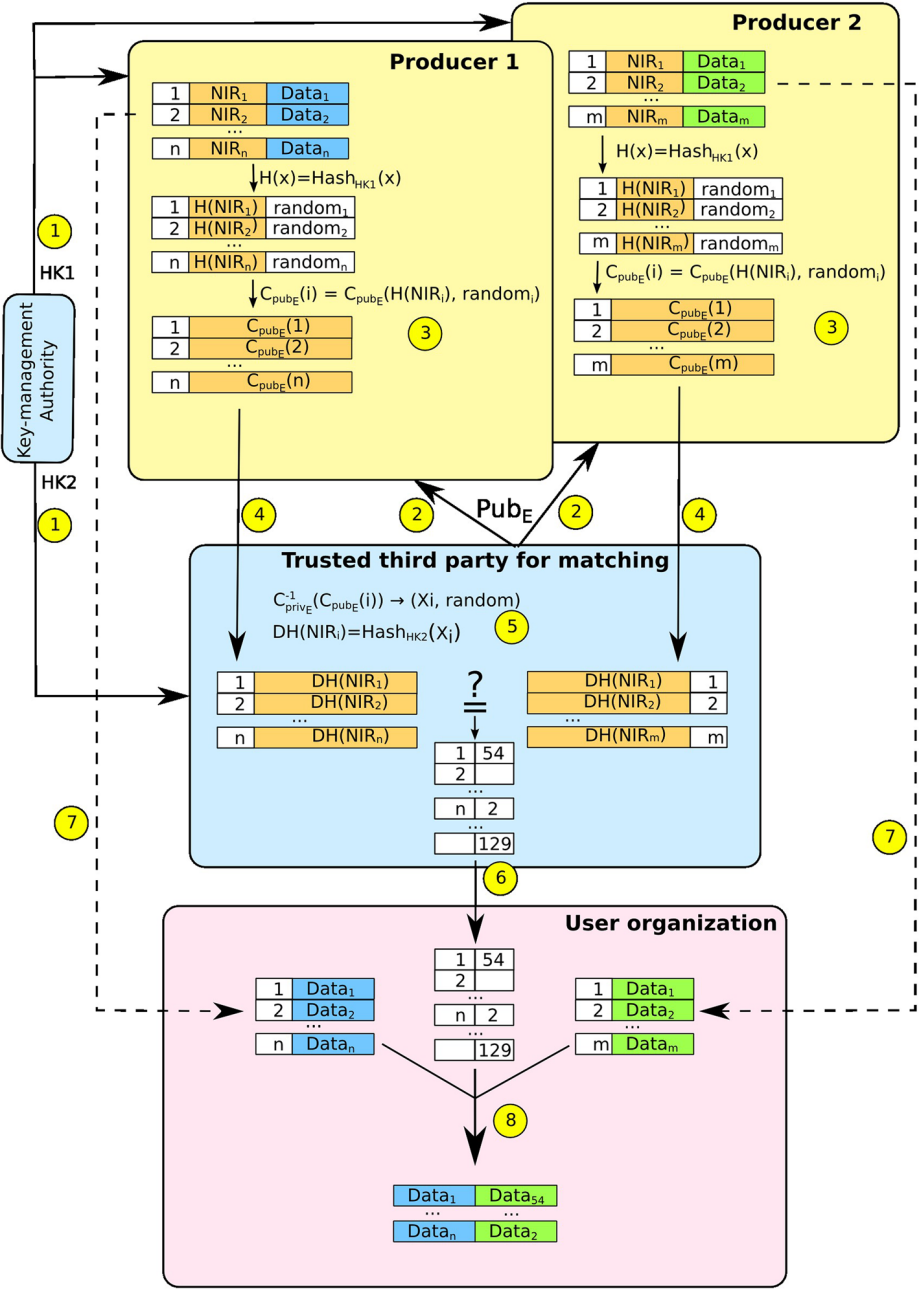
Proposed organization for secure matching

Figure 2 illustrates our proposal, with two producers of data and an organization that wishes to link the data. The numbers in yellow circles are the numbers of the steps listed below.

All of the channels of communication are supposedly secure. The linkage procedure would be as follows:

1. For each study, the key management authority sends 2 hashing keys : one (HK_1_) to the producers of the data, the other (HK_2_) to a trusted third party who will link the identifying data. Another solution could be to provide a “hashing service” to which the data to be hashed could be sent (eventually sent in a random order to diminish the risk of re-identification), but it is preferable to send as little information as possible, all the more so since the sender of information is already information : for example if an anti-cancer centre sends an NIR, it can be deduced that the corresponding person has cancer.
2. This trusted third party generates an asymmetric encryption key for the study and sends the public part (Pub_*E*_) to each producer. The key management authority is not to know this key, whose sole purpose is to ensure the secure transfer of information between the producers and organization which will link the hashed identifying data.
3. Each producer of information numbers each record sequentially (or with a unique random number for the study), so as to have an identifier that contains no information, a so-called “neutral” identifier. Each identifier field (here the NIR) is then hashed with HK_1_ provided by the key management authority. *H*(NIR) is thus obtained. A random chain, which is different for each record and has a given length, is then added to the *H*(NIR) and the whole sequence is then encrypted using Pub_*E*_, to obtain $\phantom {\dot {i}\!}C_{\text {Pub}_{E}}(H(\text {NIR}),\text {random})$.
4. Each producer sends the correspondences between $\phantom {\dot {i}\!}C_{\text {Pub}_{E}}(H(\text {NIR}),\text {random})$ and the sequential number, for each record, to the trusted third party. Even if a producer is able to obtain a file of another producer, the encryption prevents the person from linking records because of the different random part for each record, which, for the same *H*(NIR) will lead to two different results following encryption with Pub_*E*_.
5. The trusted third party receives the two files. For each record of each file, he uses Priv_*E*_ (the private part of the encryption key) to decrypt the *H*(NIR) followed by the random chain. The random chain is then removed to retain only the *H*(NIR). He then applies a second hashing with the second hashing key HK_2_ provided by the key management authority. DH(NIR) is thus obtained.
6. At this stage, the trusted third party therefore has, for each producer, a file with a correspondence between a sequential number and a DH(NIR). By comparing DH(NIR)s, the trusted third party can generate a table of correspondences between the sequential numbers of each producer of data. He sends this table to the organization authorized to link the data.
7. In addition, each producer of data sends to this organization a file containing records composed of the sequential number and the data to be linked.
8. When this organization receives these two files and the table of correspondences, it can link the data from each producer by using the sequential number of each and the table of correspondences provided.

A variation of the previous method may be needed if no Key Management Authority is available for the procedure or if one datasource contains only the NIR as the identifier and the other one only has information such as name, surname, dant and place of birth but not the NIR. In such cases, the trusted third party has to play a more central role and needs to get more identifying information than in the previous method to perform the hash function. Everything has to be done under the strict supervision of the data-protection authority (CNIL in France).

## Results

The proposed organization and workflow, while still allowing linkage between different data sources, provide strong compartmentalization of identiyfing data and other data.

Indeed, only the producers have the directly identifying information. The trusted third party only has the hashed version of this information and no other data apart from the temporary sequential number used for this study. Moreover, the trusted third party does not have the first hashing key, thus making it impossible to carry out a dictionary attack on the hashed data.

The organization wishing to link the data has no identifying information. The key management authority has no identifying or non-identifying data. It does, however, have the two hashing keys. If this authority was able to obtain the files containing the doubly hashed NIR (DH(NIR)), a dictionary attack to obtain the original NIR would be possible. It would therefore be more secure to have two different authorities each of which will generate a hashing key for the study.

Cumulation of non-identifying data can lead to a lower level of anonimity, since the more non-identifying data is gathered, the higher is the risk of re-identification (see [22] for a well-known example). The proposed method offers a strong control over who is in possession of which information, using different hashing keys for each linkage. This allows to prevent unauthorized linkage of data, to protect anonymity.

Besides, the method presented in the previous section can be generalized to any identifying information instead of the NIR. If the files to be linked do not contain any common identifier information that is specific to an individual, linkage can still be achieved on several fields such as the family name, the first name, the date and place of birth; in such cases, double-hashing and double-encryption are applied to each of these fields. The third party carrying out the linkage may then, after decryption of each field, apply a probabilistic linkage method (cf. section Probabilistic linkage), which, as we have seen, is still effective on anonymized data [12].

Unfortunately, it was not possible for us to apply the method we described in this article. Indeed, the data protection authority (CNIL) and the current legal framework do not allow public bodies to use the NIR (national unique identifier) to match datafiles, even for scientific purposes, without issuing a decree signed by the prime minister. This makes it almost impossible for researchers. The current legal framework allows private organizations to match datafiles by the NIR for scientific purposes. This is very rare and authorization from CNIL is required.

We had the opportunity to use a variation of the method described in this article for the ESPS survey (Health, health care and insurance survey) led by Irdes (Institute for Research and Information in Health Economics, private status). The aim of ESPS was to merge survey microdata, provided by a private company that carries out the survey, with administrative microdata from CNAM-TS (National Health Service in France). 8000 households (22000 persons) were interviewed to know, for example, how they perceived their health status or the reasons for not seeking care or their opinions about health.

The data matching process allowed to add the real consumption of medical services. As a trusted third party, CASD (French research data centre) received the list of identifiers of the sample : NIR, name, surname, address and generated asymmetric encryption keys for the study and a “neutral” identifier called Ben_*N*_. CASD sent Ben_*N*_, name, surname and address, encrypted using the GnuPG software, to the private company to perform the survey. CASD also hashed the NIR with a secret key known only by CNAM-TS and sent the resulting list to CNAM-TS which enriched the data with information on reimbursements for healthcare. CNAM-TS then sent the file with the enriched information and the above-mentioned “neutral” identifier Ben_*N*_ to Irdes, while the private company sent to Irdes the survey datafile with only Ben_*N*_ as the identifier, so that Irdes could match both files without getting any identifier. It was possible to add administrative microdata to the survey microdata to compare the feelings about health status with the real consumption of health services.

## Discussion

The organization that finalizes the linking of data may find itself in three situations, with two of them being non-exclusive:

- each record of producer 1 corresponds to a record of producer 2 and reciprocally; in this case linkage is total,
- there is no correspondence between some records of one producer and those of the other producer,
- there are several correspondences between some records of a one producer and those of the other producer.

The way in which the latter two situations are treated depends on the study being conducted. Incomplete linkage could be due to an error or could be perfectly normal. In the same way, multiple linkages can be treated differently depending on the objectives of the study.

To avoid the transmission of useless data, rather than each producer sending all of the data, including data that cannot be linked, the organization doing the linkage could request producers to send only data corresponding to the neutral identifiers appearing to be linked in the table of correspondences.

In addition, in Fig. 2, the organization using the linked data is the one that carries out the linking. It is possible to include another party for the final step, and thus send the linked data to the organization authorized to use them.

Concerning the key used for the first hashing (HK_1_), a possible solution would be for one of the producers to generate the key themselves and to send it to the second producer. For the second hashing key (HK_2_), the party linking the neutral identifier could generate the key. For studies carried out over long periods, however, or to ensure the reproducibility of studies (and research reproducibility in general), it would be better to conserve these keys. Indeed, in order to carry out linkage using hashed identifiers, all of the identifiers must be hashed in the same way with the same keys. For long-term studies, or studies which make use of data from previous studies, it is therefore necessary to conserve the keys used. For us, the existence of a key management authority therefore seems necessary. The issue of archiving and the use of linkage to include data from previous studies while ensuring the relative anonymity of participants is a topic of research that needs to be investigated in greater depth.

Finally, the authenticity of the keys used must be established : when an institution communicates encrypted data to another one using a public key, it must make sure that this key is well and truly the one to use to transmit data to the recipient institution and for the study in question.

If the Key-Management Authority provides the keys, it will sign them to authenticate them and encrypt them so as to ensure their confidentiality and their integrity when they are transmitted to the recipient establishments.

The data handling chain is thus made secure by encryption while the anonymity of data is ensured as the data identifiers are doubly hashed before transmission to the third party carrying out the linkage.

This research helped us in our discussions with the authorities to define a new legal framework for data matching in France. A new act, the digital act, has just been voted and will take effect before the end of 2016. A dedicated article in this law will allow public organizations to match data for scientific purposes using the NIR according to a specific process based on the method presented in this article. The law stipulates that a Key Management Authority and a trusted third party must be involved in the process as described in this article. It also stipulates that the NIR must be encrypted (“cryptographic operations” have to be performed). Instead of issuing a decree as before, only a regular authorization of CNIL will be required. This major change will foster research in many disciplines by allowing the linkage of datasources for scientific purposes.

## Conclusions

As we have seen, current computer techniques (hashing and encryption) make it possible to carry out statistical studies requiring the linkage of social and medical files while preserving a high enough level of anonymity to meet CNIL requirements.

The proposal presented above, using these techniques, would make it possible to conduct such studies more easily, more regularly and more precisely while preserving a high enough level of anonymity.

For us, it seems important to implement such a procedure, with a Key-Management Authority and the needed trusted third parties like the ones proposed here to unblock research and studies that use social and medical data. The main obstacle to setting up such a system, in our opinion, is not technical, but rather organizational in that it depends on and is made possible by the existence of a Key-Management Authority, whose role is to generate, transmit and keep the keys for each study, and trusted third parties allowing to compartmentalize information.

## Endnotes

^1^ https://en.wikipedia.org/wiki/SAFARI.

^2^ Commission Nationale de l’Informatique et des Libertés

^3^ NIR stands for “Numéro d’inscription au répertoire” ; it is a national identifier.

^4^ This situation has evolved. For example, the fiscal administration now associates the NIR with its sectorial identifier, to remove duplicates and to transmit useful fiscal information to social organizations, for example, when a welfare payment is subject to an upper limit of income.

^5^ For example, the number of different signatures produced by SHA-256 is 2^256^, a number greater than 10^77^.

^6^ For example, for words of 80 bits (10 characters each coded using one octet), the risk of collision using SHA256 is of the order of 10^−31^.

^7^ 1 byte = 8 bits = 256 possibilities.

^8^ This is the method used in ANONYMAT software, developed at Dijon CHU and validated by CNIL for the anonymization of data for linkage purposes [8].

^9^ It is nonetheless possible, before hashing, to break up information, for example, into blocks of *n* separately hashed characters, and then apply a distance calculation to these hashed blocks; the distance could be a function of the number of identical hashed blocks, or a more complex measurement using for example Bloom filters as in [23].

^10^ For files stored in an SQL-type database, a simple join query is enough to link the files.

^11^ Rather than manual validation, the descriptive information provided can also be used. Thus, in our example, the model tells us that among the 725 cases with 011, 78.66 *%* “should” be matched.

^12^ Manual validation also depends on the type of study and on the importance of the data concerned. For example, in an epidemiological study concerning the impact of a drug, we need to be as accurate as possible, and manual validation would allow us to correctly match the maximum number of records. For a less critical study, for example linking success at the baccalaureat with marks obtained during the year, a lower matching rate would be acceptable, without the need to manually verify the data at considerable expense.

^13^ Authentification can take place as follows : When Bob wants to send a message to Alice while allowing Alice to be sure that he sent the message, he applies a hash function to his message to obtain a fingerprint. Then he encrypts this fingerprint with his private key. When Alice receives the message, she can in turn apply the same hash function to the message, then decrypt the fingerprint using Bob’s public key, and finally compare the two fingerprints. If they are identical, Bob was indeed the author of the message, because only the owner of the private key (Bob) could have encrypted it in such a way that the public key (Bob’s) could decrypt it. The author of a message can thus be *authenticated*.
